# Chlorinated paraffins in hinges of kitchen appliances

**DOI:** 10.1007/s10661-021-09023-z

**Published:** 2021-04-07

**Authors:** Jannik Sprengel, Walter Vetter

**Affiliations:** Institute of Food Chemistry (170B), University of Hohenheim, Garbenstr. 28, 70593 Stuttgart, Germany

**Keywords:** Polychlorinated *n*-alkane, SCCP, MCCP, Wipe test, Lubricant

## Abstract

Chlorinated paraffins (CPs) are anthropogenic pollutants of growing environmental concern. These highly complex mixtures of thousands of homologs and congeners are usually applied as additives in lubricants or as flame retardants and plasticizers in polymers and paints. Recent studies indicated the presence of high amounts of CPs in the kitchen environment whose sources could not be unequivocally identified. One option was the use of CPs as or in lubricants of hinges. To test this hypothesis, we performed wipe tests on lubricants on 29 hinges of different types of kitchen appliances (refrigerators, baking ovens, dishwashers, freezers, microwave oven, pasta machine, food processor, steam cooker) and analyzed them for short-chain CPs (SCCPs) and medium-chain CPs (MCCPs). CPs were detected in 21 samples (72%). Per wipe, SCCP concentrations ranged between 0.02 and 10 µg (median 0.23 µg), while MCCPs ranged from 0.09 to 750 µg (median 1.0 µg). Highest MCCP amounts (380 and 750 µg per wipe, respectively) were determined in new and unused appliances. A medium correlation between SCCP content and appliance age was observed, but no additional statistic correlation between SCCP/MCCP amount and appliance type or manufacturer could be observed. CPs released from hinges by volatilization, abrasion, and cleaning processes could enter the environment and come in contact with persons living in the corresponding households.

**Supplementary Information:**

The online version contains supplementary material available at 10.1007/s10661-021-09023-z.

## Introduction

Chlorinated paraffins (CPs) are high-production volume polyhalogenated compounds whose safety is more and more disputed (Glüge et al., 2016, 2018; Zellmer et al., 2020). This highly complex group of polychlorinated *n*-alkanes is commonly subdivided according to the carbon chain length ranges which were available to producers as feedstocks from industrial petroleum hydrocarbon fractionations (Tomy et al., 1997). Specifically, polychlorinated decanes to tridecanes are termed “short-chain chlorinated paraffins (SCCPs),” polychlorinated tetra- to heptadecanes are termed “medium-chain chlorinated paraffins (MCCPs),” while those with more than seventeen carbon atoms are termed “long-chain chlorinated paraffins (LCCPs).” This differentiation according to chain length is of great importance because SCCPs were recently classified as persistent organic pollutants (POPs) by addition to Annex A of the Stockholm Convention (Conference of the Parties of the Stockholm Convention, 2017), which is connected with a ban of production and use in ratifying countries after 2017. However, the share of SCCPs was already low (~15% of the CP production volume, Glüge et al., 2016), long before the ban of SCCPs was initiated. As of today, virtually no restrictions exist for the use of MCCPs and LCCPs.

Due to their persistence and lipophilicity, CPs were identified in environmental samples all over the world (Fridén et al., 2011; Krätschmer et al., 2019; Yuan et al., 2017b; Zeng et al., 2011; Zhou et al., 2020). However, several findings indicated that human exposure to CPs must not necessarily originate from the intake of contaminated food. For instance, high CP levels collected via wipe tests in household kitchens indicated a widespread occurrence although the sources could not be identified (Bendig et al., 2013; Gallistl et al., 2017). Moreover, other studies showed that baking ovens and hand blenders were containing CPs (Gallistl et al., 2018; Yuan et al., 2017). However, further potential sources of CPs were likely to exist in urban environments including kitchens. One potential opportunity could be CP-containing lubricants on hinges.

The goal of this study was to collect wipe tests from hinges from several kitchen appliances and analyze them for the possible presence of CPs. Samples were taken by wipe tests and extracted according to Gallistl et al. (2018). Sample extracts were released from matrix by means of sulfuric acid (Bendig et al., 2013), and SCCPs and MCCPs were quantified by means of gas chromatography with electron capture negative ion mass spectrometry conducted in the selected ion monitoring mode (GC/ECNI-MS-SIM), which is one of the most frequently used methods as of today (Table S1). However, we also opted to improve the precision of this setup by using single chain CP mixtures as reference standards (Sprengel & Vetter, 2019).

## Materials and methods

### Chemicals and standards

*n*-Hexane (for pesticide residue analysis grade) was purchased from Th. Geyer (Renningen, Germany). 2,2,4-Trimethylpentane (*i*-octane, for pesticide residue analysis grade) was bought from Fluka Analytics (Seelze, Germany). Sulfuric acid (96–98%, *p.a*.) was obtained from Carl Roth (Karlsruhe, Germany). Sodium sulfate (> 99%, water free, *p.a*.) and silica gel 60 (for column chromatography grade) were ordered from Sigma-Aldrich (Seelze, Germany). Perdeuterated α-hexachlorocyclohexane (α-PDHCH) was used as a recovery standard and has been synthesized in our work group (Vetter & Luckas, 1995). 6´-MeO-BDE 66 (BCIS), also synthesized in our work group (Vetter et al., 2011), was used as instrumental standard and added to standard and sample solutions prior to injection. Technical CP mixtures at 100 ng/µL were from Dr. Ehrenstorfer (Augsburg, Germany) and were additionally diluted to 10 ng/µL for quantification. Single-chain CP mixtures of C_10_- to C_17_-CPs (Table S2) were synthesized in our working group (Sprengel & Vetter, 2019; Sprengel et al., 2019).

### Sample preparation

During 2018, *n* = 29 hinges of kitchen appliances were sampled by wipe tests in private households in Southern Germany. Screened samples included nine refrigerators (R1–R9), seven baking ovens (B1–B7), five dish washers (D1–D5), four freezers (F1–F4) as well as one microwave oven, one steam cooker (SC), one pasta machine (PM), and one food processor. Additionally, seven known manufacturers were indicated by lowercase letters “a–g” in terminal position of the codes while unknown manufacturers were labeled “x” (e.g., R1a). In each case, a cotton wipe was wet with ~ 2 mL *n*-hexane and wiped slowly and with slight pressure over the hinges of the respective appliances. Since only the accessible external part of the hinges could be wiped, the absolute amount of lubricant used in the corresponding hinges could not be determined. With the exception of the pasta machine, all appliances were wiped only once to preserve function. Loaded wipes were stored in sealed amber glass vials (30 mL) at −18 °C until analysis. The sample cleanup followed the procedure described by Gallistl et al. (2018). In brief, wipes were extracted with *n*-hexane, and the extract was weighed (Table S3, SI). For the new and unused appliances B1 and PM, the extract was assumed to originate only from wiped lubricant. In used appliances, however, the sample weight must not necessarily consist of only lubricants, because other deposits like fat, dust, and other particles also contribute to the weighed amounts. Hence, analytical results will be discussed based on absolute CP amounts in the samples (µg/wipe). The lipophilic matrix residue was destroyed with sulfuric acid and a subsequent cleanup by adsorption chromatography using silica gel (Bendig et al., 2013). Sample solutions were brought to ~ 100 µL under a gentle air stream for determination of the recovery and a qualitative estimate of CP levels. For CP quantification, sample solutions were diluted to 10–100 ng/µL, if needed, to match the concentration of the standards. Recovery rates of the internal standard α-PDHCH were 97 ± 26%.

### Instrumentation

CPs were determined by gas chromatography coupled with electron capture negative ion mass spectrometry (GC/ECNI-MS) using an Agilent 7890/5975C system (Agilent, Waldbronn, Germany). Separations were performed on a 30 m × 0.25 mm i.d., 0.25 µm d_f_ Optima 5 MS (Macherey–Nagel, Düren, Germany) using the oven program of Gallistl and Vetter (2016). GC/ECNI-MS determinations were performed in selected ion monitoring (SIM) mode. CP quantification followed methods described elsewhere (Reth et al., 2005; Sprengel & Vetter, 2019). In short, the sum of the relative peak area of the [M-Cl]^−^ fragment ions of the Cl_4_- to Cl_14_-homologs was correlated exponentially with the calculated chlorine content. However, instead of SCCP/MCCP mixtures, self-synthesized single-chain CP mixtures were used to create calibration curves for each chain length separately. Additionally, correction factors for different degrees of chlorination were applied as described by Mézière et al. (2020). The reported values were modified by taking into account increasing response in GC/ECNI-MS in the range Cl_6_–Cl_10_, and strongly depleting responses at low (< Cl_6_) and very high Cl degree (> Cl_10_) (Reth et al., 2005; Yuan et al., 2017). Additionally, slight response differences between the chain lengths were observed and taken into account, creating a matrix of correction factors depending on the chain length and chlorine content of the homolog (Table S4). Each correction factor was multiplied with the respective peak area to account for response differences. The relative differences between calculated and actual chlorine contents of the standards used were always below 5%. Quantification of two technical CP standards (SCCP 55.5% Cl (C_10_- to C_13_-CPs) and MCCP 52% Cl (C_14_- to C_17_-CPs)) at 10 and 100 ng/µL resulted in a very good match of measured and actual amount (110 ± 7 and 104 ± 2%, respectively).

### Quality assurance

All glassware was rinsed with detergent, demineralized water, acetone, and distilled cyclohexane/ethyl acetate (46:54, w/w) prior to use. For each batch of 10 wipe tests, a procedural blank and a cotton pad blank each were performed. Traces of C_10_-, C_11_-, and C_14_-CPs were subtracted as relative peak areas from the samples, and only samples with at least 10 times the blank levels were considered for quantification. Limits of quantification (LOQ) and detection (LOD) were concentrations which gave a signal of the most abundant homolog group with a signal-to noise ratio of 10 and 3, respectively. Qualitative verification was achieved through rigorous comparison of retention time and isotope ratio as described previously (Sprengel & Vetter, 2019).

### Statistical analysis

All statistical tests were performed with IBM SPSS Statistics 26 (Armonk, NY, USA). A Kolmogorov-Smirnov test on the SCCP and MCCP amounts showed that the absolute CP amounts were not normally distributed. Therefore, non-parametric tests were used for statistical analyses. Samples from manufacturer g and f (both *n* = 1) as well as the two samples of unknown manufacturers x were not included for Spearman´s correlation and Kruskal-Willis’s tests concerning the manufacturer.

## Results and discussion

CPs were detected in wipes of 21 of 29 samples (detection frequency (DF) = 72%, Table 1). SCCPs and MCCPs showed similar DF (19 vs. 18 positive samples or 66% vs. 62% for SCCPs and MCCPs, Table 1). Sixteen samples featured both SCCPs and MCCPs albeit at varying ratios (Table 1). Accordingly, only three samples contained solely SCCPs and two just MCCPs.

**Table 1:** CP amounts and detection frequencies (DF) in wipe tests from hinges of 29 kitchen appliances. *nd* not detected (< 0.01 µg); *nq* not quantifiable. For the calculation of mean/median values, nd/nq were set as 0.5*MLD (0.01 µg)

			SCCPs		MCCPs		∑CP	
Appliance	Sample	Purchase year	Amount (µg)	Chlorination degree (%)	Amount (µg)	Chlorination degree (%)	Amount (µg)	SCCP/MCCP ratio
Refrigerator *n* = 9	R1a	2008	0.60	56.8	2.3	56.9	2.9	0.26
	R2x	2011	0.20	63.3	1.0	54.2	1.2	0.19
	R3c	2015	0.87	65.5	1.1	55.6	2.0	0.77
	R4d	2003	0.77	56.0	nd	–	0.77	–
	R5e	2003	0.28	58.4	0.15	51.3	0.43	1,86
	R6a	2014	0.14	54.4	0.18	51.3	0.33	0.77
	R7x	2003	0.04		nd	–	–	–
	R8a	2015	nd	–	nd	–	–	–
	R9a	2017	nd	–	nd	–	–	–
	Mean/median		0.30/0.20 (DF = 78%)		0.54/0.15 (DF = 56%)		0.86/0.33 (DF = 78%)	
Baking oven *n* = 7	B1a^a^	2018	nd	–	380	52.7	380	–
	B2a	2016	0.07	61.2	4.2	53.1	4.3	0.02
	B3a	2008	nq^b^	71.6	2.5	55.3	2.5	–
	B4a	2017	0.23	55.8	0.93	49.1	1.2	0.25
	B5b	2015	0.17	61.3	0.96	53.6	1.1	0.18
	B6a	2007	nd	–	0.09	51.7	0.09	–
	B7a	2017	nd	–	nd	–	–	–
	Mean/median		0.06/0.01 (DF = 57%)		48/0.94 (DF = 86%)		48/1.1 (DF = 86%)	
Dishwasher *n* = 5	D1a	2008	0.39	58.8	160	48.7	160	0.00
	D2b	2015	0.02	61.6	6.1	51.1	6.1	0.00
	D3d	2006	0.63	56.3	0.51	56.3	1.1	1.23
–	D4a	2016	0.49	59.3	nd	–	0.49	–
	D5a	2017	nd	–	nd	–	–	–
	Mean/median		0.31/0.39 (DF = 80%)		34/0.51 (DF = 60%)		34/1.1 (DF = 80%)	
Freezer *n* = 4	F1b	2015	0.80	62.7	1.3	55.5	2.1	0.61
	F2a	2014	0.18	55.0	0.13	51.1	0.31	1.34
	F3e	2016	nd	–	nd	–	–	–
	F4e	2006	nd	–	nd	–	–	–
	Mean/median	5.3/3.5	0.25/0.09 (DF = 50%)		0.37/0.07 (DF = 50%)		0.62/0.16 (DF = 50%)	
Microwave oven (a)		2008	nd	–	nd	–	–	–
Steam cooker (SC) (a)		2006	0.49	59.3	2.4	51.1	2.9	0.21
Pasta machine (PM)^a^ (f)		2003	10	58.8	750	55.6	760	0.01
food processor (g)		2007	nd	–	nd	–	–	–
All *n* = 29	Mean/median		0.79/0.23 (DF = 66%)		62/1.0 (DF = 62%)		63/1.2 (DF = 72%)	

^a^Unused appliance

^b^Baking oven contained SCCPs with >70% Cl, which could not be quantified, but gave large signals

The production dates of at least 24 samples definitely predated classification of SCCPs as POPs (Conference of the Parties of the Stockholm Convention, 2017), which might explain the high DF of SCCPs. In accordance with the ban of SCCPs in the European Union, the only sample (B1a) manufactured after 2017 did not contain any measurable amount of SCCPs (Table 1). Noteworthy, several SCCP-containing products were produced after the ban of SCCPs in Europe in 2012 (European Parliament, Council of the European Union, 2012). However, it could not be unequivocally determined if the SCCP share in the lubricant exceeded the 0.15% that is legally allowed (European Parliament, Council of the European Union, 2019). A moderate but significant correlation between appliance age and SCCP concentration (Spearman’s *ρ* = 0.470, *p* = 0.042) supported this hypothesis (Fig. S1). However, MCCP amounts (0.09–750 µg/wipe; median 1.0 µg/wipe) were generally higher than those of SCCPs (0.02–10 µg/wipe; median 0.23 µg/wipe), with only eight samples containing less than 1 µg/wipe MCCPs compared to one sample featuring > 1 µg/wipe SCCPs (Table 1). However, SCCP/MCCP ratios in samples with both CP classes were not uniform among appliance types and manufacturers, and a Kruskal-Wallis test indicated no significant impact (*p* > 0.05) of appliance type or manufacturer (which are usually producing in different countries) on SCCP or MCCP amount, chlorination degree, or SCCP/MCCP share.

The highest amounts of both SCCPs (10 µg) and MCCPs (750 µg) were detected in wipe tests of a 20-year-old but unused pasta machine (Table 1). In this case, CPs contributed with ~ 5% to the lubricant mass collected by the wipe (15 mg). The use of MCCPs in lubricants in the EU has been reported for at least three decades (European Chemicals Bureau, 2005). A second wipe test of sample PM gave ~ 19% of the CP amount of the first wipe of the hinges, contributing again with 5% to the sample weight. Hence, it was evident that a high share of CP-containing lubricant could be collected by the wipe tests.

According to the SCCP shares to the sum of CPs in CP-positive hinges, the samples could be separated in two groups:

- Group 1: < 20% SCCP (B1a-B6a, D1a, D2b, R2x, SC, PM)
- Group 2: > 20% SCCP (R1a, R3c-R7x, D3d, D4a, F1b, F2a)

**Fig. 1:**
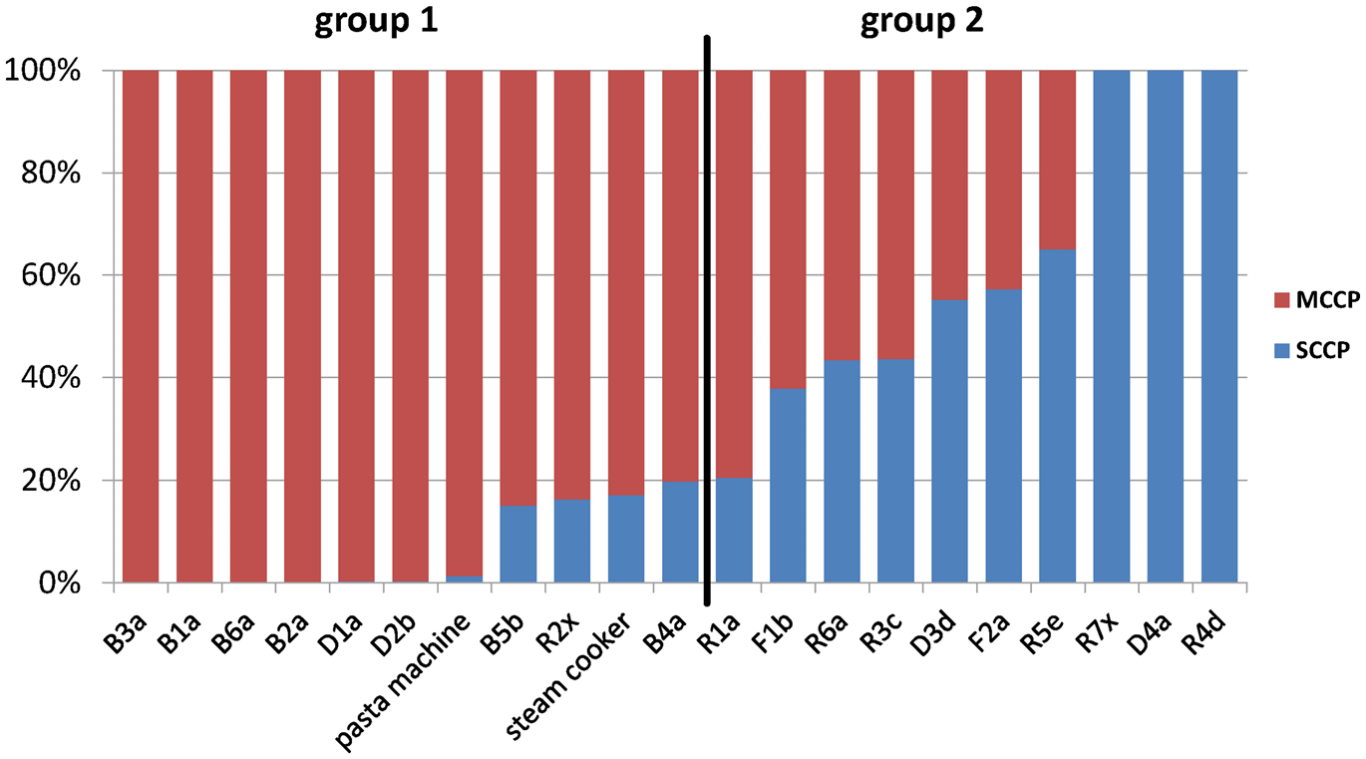
SCCP/MCCP-ratios of the CP-positive wipe tests of 21 kitchen appliances

The low SCCP shares of group 1 could originate from impurities in MCCP formulations (Fig. 1). For example, Chinese CP formulations are rather classified by chlorination degree (e.g., 52% chlorine in CP-52 products) than by chain length ranges (Glüge et al., 2018). Chinese technical CP products consist of three major classes, i.e., 42% (CP-42), 52% (CP-52), and 70% (CP-70), respectively. In the present samples, the Cl content of MCCPs was 48.7–56.9% (Table 1). Apart from two samples with chlorine contents of < 51%, this range was close to the %Cl content determined for the 52% Cl MCCP mixture (54.3% Cl). CP-52 formulations have been shown to contain highly varying amounts of SCCPs (Gao et al., 2012, 2016; Li et al., 2018; Sprengel & Vetter, 2020). Low shares of SCCPs (i.e., 4% and 8%, respectively) were previously detected in two CP-52 products, mainly consisting of MCCPs (Sprengel & Vetter, 2020). Arguably, the CP formulations in most appliances were CP-52 with low SCCP shares, and higher Cl degrees could indicate the partial release of more volatile CPs with low(er) Cl degree. For instance, Gallistl et al*.* suggested that CPs applied in baking ovens could be released and transferred into the prepared food or into the air (Gallistl et al., 2018). Recently, it was shown that CPs can be volatilized from its source in a baking oven environment (Perkons et al., 2019). However, CP mixtures with lower chlorine contents than 52% were probably applied in appliances B4a and D1a. Noteworthy, the %Cl content of SCCPs (54.4–71.6%) was generally higher than in MCCPs (Table 1).

Baking ovens were the only appliance type exclusively containing low SCCP shares (group 1). They also showed the highest overall DF of CPs (86%, Table 1) and were the only product group where MCCPs showed a higher DF (86%) than SCCPs (DF = 57%). Similarly, a previous study conducted on baking oven doors showed that MCCPs were much more prominent (Gallistl et al., 2018). Accordingly, SCCP amounts found in baking ovens were among the lowest (0.07–0.23 µg/wipe) of all samples. However, the MCCP amounts varied by four orders of magnitude (Table 1). The maximum MCCP amount in baking ovens of 380 µg/wipe was detected in a new and unused instrument (B1a), which corresponded to ~ 14% of the wiped lubricant (2.6 mg lubricant mass). The chlorine content of 52.6% in sample B1a indicated the use of a CP-52 product. Other baking oven samples showed much lower MCCP amounts (median value: 0.94 µg MCCPs/wipe), which could indicate a release of CP containing lubricant during usage and/or cleaning processes. However, Spearman’s, Kruskal-Willis’s, and Kendall-Tau’s correlation tests between chlorine content and appliance age did not support this hypothesis (*p* >> 0.05 in all cases).

In contrast to group 1, SCCP amounts in group 2 samples were too high (> 20%) to label them as impurities in MCCP feedstocks (max. 1% of the CP formulation, European Parliament, Council of the European Union, 2019). These samples indicated an intentional application of SCCP-containing formulations. Possibly, CP stocks containing both SCCPs and MCCPs as has been reported for several Chinese CP products (Gao et al., 2012, 2016; Li et al., 2018) were used in these cases. Irrespective of these uncertainties, it is not advisable to determine only SCCPs or MCCPs or LCCPs in samples, because they frequently co-occur in environmental samples.

**Fig. 2:**
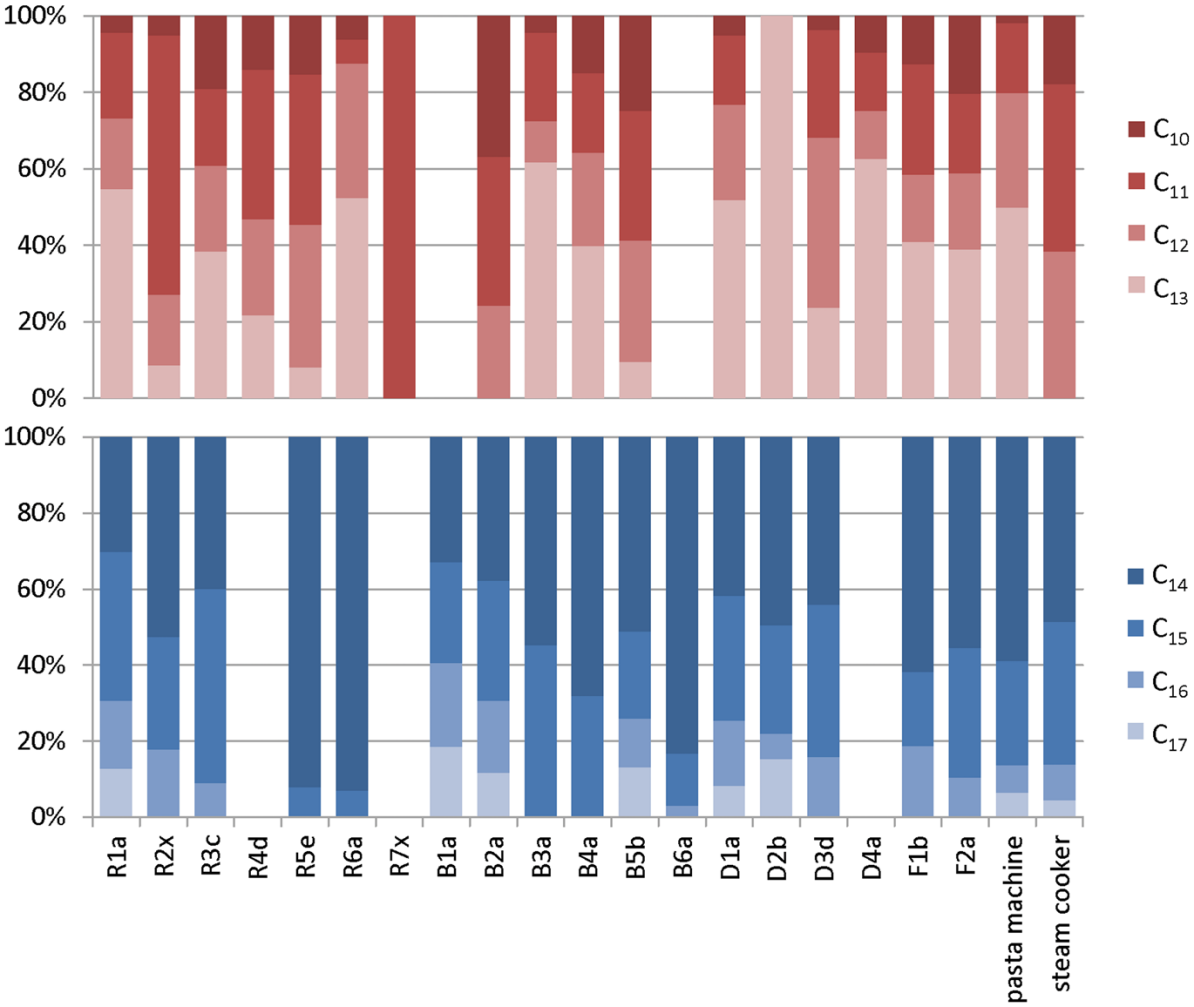
Chain length distributions of the SCCPs and MCCPs found in wipe tests from hinges of 21 kitchen appliances

Interestingly, wipes of CP-containing hinges of freezers (F1b, F2a) and all CP-containing refrigerators (exception: R2x) showed high shares of SCCPs. This indicated a preferred use of shorter CP chain lengths in lubricants of cooling appliances (Fig. 2).

CP patterns are strongly depending on the analytical method, and comparisons between different studies are difficult to draw (Mézière et al., 2020; van Mourik et al., 2016). However, comparison of CP patterns of samples within the present study seemed to be appropriate. SCCPs were dominated by varying ratios of C_11_- > C_12_- and/or C_13_-CPs (Fig. 2), reflecting the heterogeneous nature of technical SCCP products (Li et al., 2018; Yuan et al., 2017a). However, the small C_10_-CP shares in all samples were in agreement with the commercial mixtures analyzed in the previous studies. C_14_-CPs were, with the exception of sample R1a, the dominant MCCP homolog group (Fig. 2), again corresponding to compositions of MCCP mixtures (van Mourik et al., 2016; Yuan et al., 2017b).

The varying CP homolog patterns produced further evidence that these may be influenced by consumer usage habits (frequency and mode of usage, cleaning), because it appeared unlikely that such a high number of different technical CP products had been applied in the past, despite the wide range of appliance types and production years. However, the CP pattern in two subsequent wipes of the pasta machine was nearly identical (Fig. S2). Hence, changes in the CP patterns by mechanical cleaning were regarded to be rather minimal, but they could gradually emerge after years of use. One wipe of a baking oven hinge (B3a), however, stood out due to its very high SCCP chlorine content of 71.6% Cl. This indicated the use of a highly chlorinated CP mixture, since alteration of a short-chain CP-52 mixture (e.g., by volatilization or transformation) would have required a shift from on average ~ 5 to ~ 9 chlorine atoms per molecule which seemed unlikely.

Several of the kitchen appliances discussed so far could directly expose consumers by releasing CPs. Similarly, dishwashers could transfer CPs onto the cleaned utensils and into wastewater. Also, the lubricant of the pasta machine would come in direct contact with the dough during use, which could lead to the contamination of food, similarly to observations made with hand blenders (Yuan et al., 2017b). Last but not least, regular cleaning of the kitchen (usually with dishcloths or sponges) could partly remove CPs together with lubricant (see above). For instance, up to 55 µg CPs were determined in initially CP-free dishcloths after a 2-week use (Gallistl et al., 2017). Considering the high CP amounts in two unused appliances, especially new and previously unused appliances, could represent a hitherto overlooked source of CP exposure. Vice versa, it could not be excluded that used appliances were initially richer in CPs (i.e., similarly high as the new ones in this study). These diffuse sources could lead to human exposure and/or environmental contamination with CPs prior to disposal of the appliances. Additionally, since 66% of the sampled appliances contained POPs (SCCPs), the eventual disposal of many kitchen appliances in the future could be greatly complicated.

## Conclusions

Wipe tests enabled us to show that CPs were present in 72% of hinges of kitchen appliances. We also established an improved quantification method using single chain length CP standards, allowing for more specific and precise CP determinations. Although no correlation between appliance, manufacturer, or age and CP amount could be established, the exceptionally high amounts in two unused appliances indicated a release of CPs over time. This previously unknown CP source may lead to increased exposure for the consumer.

## Supplementary Information

Below is the link to the electronic supplementary material.

Supplementary file1 (DOCX 100 KB)
