# Supplementary material for: Chlorinated paraffins in hinges of kitchen appliances
Source: Environ Monit Assess. 2021 Apr 7;193(5):250. doi: 10.1007/s10661-021-09023-z (PMC8026443; doi:10.1007/s10661-021-09023-z)
Supplement: Supplementary file 1 — Supplementary file1 (DOCX 100 KB) [file 10661_2021_9023_MOESM1_ESM.docx]

Supplementary Information for

**Chlorinated paraffins in hinges of kitchen appliances**

Jannik Sprengel and Walter Vetter*

University of Hohenheim, Institute of Food Chemistry (170b), Garbenstr. 28, D-70593 Stuttgart, Germany

* corresponding author

Walter Vetter

University of Hohenheim

Institute of Food Chemistry

Garbenstraße 28

D-70599 Stuttgart, Germany

E-mail: walter.vetter@uni-hohenheim.de

**Tab. S1.** Research papers on CP analysis according to SCOPUS database, distributed by analytical method and CP species (S/M/LCCP) as of October 27^th^, 2020.

| method | 2016 | 2017 | 2018 | 2019 | 2020 | sum |
| --- | --- | --- | --- | --- | --- | --- |
| GC/ECNI-LRMS | 13 (13/4/0) | 16 (16/7/0) | 13 (13/7/0) | 18 (18/9/0) | 14 (14/7/1) | 74 (74/34/1) |
| APCI-qTOF-MS | 1 (1/1/0) | 5 (5/4/4) | 4 (4/4/4) | 7 (7/7/6) | 8 (8/8/6) | 25 (25/24/20) |
| GC/qTOF-MS | 1 (1/1/0) | 6 (6/2/0) | 5 (5/5/0) | 4 (4/4/0) | 5 (5/5/0) | 21 (21/17) |
| LC/qTOF-MS | 1 (1/0/0) | 2 (2/2/1) | 2 (2/2/1) | 6 (6/6/3) | 3 (3/3/2) | 14 (13/7) |
| GCxGC/qTOF-MS | 3 (3/3/0) | 4 (4/3/0) | 4 (4/2/0) | 2 (2/2/1/0) | 0 | 13 (13/10/0) |
| GC/ECNI-HRMS^a^ | 1 (1/1/0) | 0 | 2 (2/1/0) | 3 (3/2/1) | 2 (2/2/0) | 8 (8/6/1) |
| GCxGC/ECNI-LRMS | 0 | 0 | 1 (1/0/0) | 3 (3/2/0) | 3 (3/3/0) | 7 (7/5/0) |
| GC/MSxMS | 0 | 1 (1/0/0) | 1 (1/0/0) | 1 (1/0/0) | 1 (1/1/0) | 4 (4/0/0) |
| GCxGC/MSxMS | 0 | 0 | 0 | 0 | 3 (3/3/0) | 3 (3/3/0) |
| LC/Orbitrap-HRMS | 0 | 0 | 0 | 0 | 2 (2/2/0) | 2 (2/2/0) |
| GC/ECD | 1 (1/1/0) | 0 | 1 (1/0/0) | 0 | 0 | 2 /2/1/0) |
| others^b^ | 1 (1/1/0) | 3 (3/1/0) | 1 (1/0/0) | 1 (1/1/0) | 1 (1/1/0) | 7 (7/4/0) |
| sum | 21 (21/11/0) | 37 (37/19/5) | 34 (34/20/5) | 45 (45/32/10) | 43 (43/36/10) | 180 (180/118/30) |

^a^ sum of Orbitrap- and sector field HRMS instruments

^b^ other methods were only described once each, including GCxGC/ECD, GC/FID, GPC/GC/ECNI-LRMS, MALDI-TOF-MS, planar chromatography, APCI-ECNI-HRMS and LC/MSxMS

**Tab. S2.** Single chain CP standards used for quantification, and their chlorine content as determined by EA.

| Chain length | Chlorine content |
| --- | --- |
| C_10_-CPs | 53.8% |
|  | 58.0% |
|  | 60.5% |
|  | 64.7% |
| C_11_-CPs | 51.2% |
|  | 56.2% |
|  | 62.6% |
|  | 68.5% |
| C_12_-CPs | 51.9% |
|  | 59.0% |
|  | 59.9% |
|  | 67.1% |
| C_13_-CPs | 50.3% |
|  | 54.4% |
|  | 59.6% |
|  | 66.4% |
| C_14_-CPs | 50.1% |
|  | 52.9% |
|  | 53.6% |
|  | 67.3% |
| C_15_-CPs | 50.9% |
|  | 53.5% |
|  | 55.0% |
|  | 68.5% |
| C_16_-CPs | 48.6% |
|  | 50.9% |
|  | 53.1% |
|  | 58.9% |
| C_17_-CPs | 50.3% |
|  | 54.9% |
|  | 61.3% |

**Tab. S3.** Weight of the extract from wipe tests from hinges of 29 kitchen appliances as well as dilution factor used for the sample solution.

| **Sample** | **extract [mg]** | **dilution factor** |
| --- | --- | --- |
| R1a | 11.4 | 5 |
| R2x | 19.6 | 1 |
| R3c | 2.7 | 1 |
| R4d | 8.2 | 1 |
| R5e | 3.0 | 1 |
| R6a | 2.7 | 1 |
| R7x | 2.1 | 1 |
| R8a | 5.2 | 1 |
| R9a | 1.5 | 1 |
| B1a | 2.6 | 10 |
| B2a | 20.3 | 1 |
| B3a | 3.1 | 1 |
| B4a | 25.4 | 1 |
| B5b | 0.9 | 1 |
| B6a | 0.9 | 1 |
| B7a | 1.1 | 1 |
| D1a | 5.7 | 10 |
| D2b | 18.2 | 5 |
| D3d | 10.4 | 1 |
| D4a | 16.6 | 1 |
| D5a | 3.0 | 1 |
| F1b | 2.8 | 1 |
| F2a | 2.9 | 1 |
| F3e | 1.0 | 1 |
| F4e | 1.2 | 1 |
| pasta machine (f) | 15.1 | 20,000 |
| steam cooker (a) | 4.6 | 10 |
| food processor (g) | 1.0 | 1 |
| microwave oven (a) | 0.8 | 100 |

**Tab. S4.** Empirically determined correction factors for C_10_- to C_17_-chlororparaffin (CP) homologs.

|  | C_10_ | C_11_ | C_12_ | C_13_ | C_14_ | C_15_ | C_16_ | C_17_ |
| --- | --- | --- | --- | --- | --- | --- | --- | --- |
| Cl_4_ | 25 | 25 | 25 | 25 | 25 | 25 | 25 | 25 |
| Cl_5_ | 2 | 5 | 5 | 5 | 5 | 5 | 5 | 5 |
| Cl_6_ | 0.5 | 1 | 1 | 1 | 1 | 1 | 1 | 1 |
| Cl_7_ | 0.25 | 0.5 | 0.5 | 0.5 | 0.5 | 0.5 | 0.5 | 0.5 |
| Cl_8_ | 0.5 | 0.25 | 0.25 | 0.25 | 0.5 | 0.5 | 0.5 | 0.5 |
| Cl_9_ | 1 | 0.5 | 0.5 | 0.5 | 0.5 | 1 | 1 | 1 |
| Cl_10_ | 1 | 1 | 1 | 1 | 1 | 2 | 2 | 2 |
| Cl_11_ | 2 | 2 | 2 | 2 | 2 | 20 | 20 | 50 |
| Cl_12_ | 10 | 10 | 10 | 10 | 20 | 100 | 100 | 200 |
| Cl_13_ | - | 20 | 20 | 100 | 100 | 200 | 200 | 500 |
| Cl_14_ | - | 50 | 100 | 200 | 200 | 500 | 500 | 500 |

**Fig. S1.** SCCP/MCCP amounts in wipe tests of hinges of 21 kitchen appliances in correlation to appliance age.

**pasta machine wipe #1**

**pasta machine wipe #2**

**MCCPs**

**MCCPs**

**SCCPs**

**SCCPs**

**Fig. S2.** CP homolog patterns of two subsequent wipe tests of a pasta machine. Although the second wipe only gave ~15% of the CP amount of the first wipe, the homolog patterns remained very similar.
